# Copper chelation selectively kills colon cancer cells through redox cycling and generation of reactive oxygen species

**DOI:** 10.1186/1471-2407-14-527

**Published:** 2014-07-21

**Authors:** Maamoun Fatfat, Raghida Abou Merhi, Omar Rahal, Detcho A Stoyanovsky, Angela Zaki, Hazar Haidar, Valerian E Kagan, Hala Gali-Muhtasib, Khaled Machaca

**Affiliations:** 1 Department of Biology, American University of Beirut, Beirut, Lebanon; 2 Department of Biology, Lebanese University, Beirut, Lebanon; 3 Department of Environmental and Occupational Health, University of Pittsburgh, Pittsburgh, USA; 4 Department of Physiology and Biophysics, Weill Cornell Medical College, Doha, Qatar

**Keywords:** Colon cancer, Metal chelation, TPEN, Redox cycling, Copper, Reactive oxygen species

## Abstract

**Background:**

Metals including iron, copper and zinc are essential for physiological processes yet can be toxic at high concentrations. However the role of these metals in the progression of cancer is not well defined. Here we study the anti-tumor activity of the metal chelator, TPEN, and define its mechanism of action.

**Methods:**

Multiple approaches were employed, including cell viability, cell cycle analysis, multiple measurements of apoptosis, and mitochondrial function. In addition we measured cellular metal contents and employed EPR to record redox cycling of TPEN–metal complexes. Mouse xenografts were also performed to test the efficacy of TPEN in vivo.

**Results:**

We show that metal chelation using TPEN (5μM) selectively induces cell death in HCT116 colon cancer cells without affecting the viability of non-cancerous colon or intestinal cells. Cell death was associated with increased levels of reactive oxygen species (ROS) and was inhibited by antioxidants and by prior chelation of copper. Interestingly, HCT116 cells accumulate copper to 7-folds higher levels than normal colon cells, and the TPEN-copper complex engages in redox cycling to generate hydroxyl radicals. Consistently, TPEN exhibits robust anti-tumor activity *in vivo* in colon cancer mouse xenografts.

**Conclusion:**

Our data show that TPEN induces cell death by chelating copper to produce TPEN-copper complexes that engage in redox cycling to selectively eliminate colon cancer cells.

## Background

Cancer is one of the leading causes of mortality and represents a tremendous burden on patients and societies. Colorectal cancers are associated with one of the highest morbidity and mortality rates in both men and women (Globocan 2008, IARC 2010). Although the etiology of cancer varies greatly between different types of neoplasms, a hallmark finding is a defect in cell cycle regulation [1,2]. Such cell cycle disruption is associated with genomic instability, due to mutations and chromosomal aberrations that disrupt critical housekeeping functions, including DNA repair and cell cycle checkpoints. Because these homeostatic mechanisms are essential for both normal and cancerous cells, treatments targeted at selectively destroying cancer cells become challenging.

The G2-M transition marks the entry of cells into the division phase of the cell cycle. Interestingly, metal chelation using the acyclic amino metal chelator *N*, *N*, *N*’, *N*’-tetrakis-[2-pyridylmethyl]-ethylenediamine (TPEN) prevents frog oocytes from entering meiosis [3]. TPEN, an uncharged polydentate ligand with nitrogens as donor atoms, has remarkably high affinity for a broad spectrum of metal ions, including copper, iron and zinc [4]. TPEN-induced meiotic arrest is due to the lack of activation of Cdc25C, a dual specificity phosphatase in oocytes that represents the rate limiting step in activating the master regulator of the G2-M transition cyclin-dependent kinase 1 (Cdk1) [5]. This is because Cdc25C is a Zn^2+^-binding protein and removal of Zn^2+^ inhibits its ability to interact with and dephosphorylate Cdk1 [3]. TPEN treatment also results in meiosis arrest in mouse oocytes [6].

TPEN-dependent metal chelation has noticeably distinct effects during the mitotic cell cycle as compared to meiosis. TPEN induces apoptosis in different cell types, including lymphocytes and splenocytes [7,8], epithelial cells [9], hepatocytes [10], breast cancer [11], HT-29 colorectal cancer [12], ovarian cancer [13], pancreatic cancer [14], and prostate cancer [15]. However the mechanisms of actions proposed for the TPEN-dependent cell killing are quite varied: TPEN was proposed to decrease the levels of the apoptosis inhibitor XIAP due to Zn^2+^ chelation [15]. Zn^2+^ depletion has also been implicated in mitochondrial injury, activation of caspases (primarily caspase 3) and apoptosis [8,9,14]. Furthermore, TPEN treatment results in depletion of glutathione causing increased redox stress [10]. Collectively these findings argue that TPEN induces cell killing through varied mechanisms, which may be expected given the diverse roles that metals chelated by TPEN play in physiological processes. Indeed TPEN has been used as a selective Zn^2+^ chelator despite the fact that it has a significantly higher affinity for Cu^2+^ (Stability constant for Zn 15.5 and for Cu 20.5) [16]. There is evidence at least in the hippocampus that TPEN *in vivo* chelates Zn^2+^ with better efficiency as compared to Cu^2+^[17].

Metal homeostasis is important for biological function and needs to be tightly regulated since either metal deficiencies or metal excesses tend to be toxic. Metals have played important roles in cancer treatment since ancient times with the use of arsenic trioxide to treat different cancers including leukemia in the 18^th^ and 19^th^ century [18]. More recently platinum based compounds such as cysplatin and carboplatin have become the chemotherapeutic agents of choice for many cancers [19]. Interestingly cancer cells are addicted to high iron levels and accumulate the metal through transferrin-dependent uptake [20,21]. Furthermore cancer cells concentrate high levels of copper, which is presumed to be important for both angiogenesis and metastasis [22]. Therefore, transition metals are likely to play important roles in the development and growth and neoplasms.

Here we show that TPEN-mediated metal chelation results in selective killing of HCT116 colon cancer cells without affecting normal cells. TPEN cytotoxicity is due to the generation of ROS as it is reversed by antioxidants. Interestingly, HCT116 colon cancer cells accumulate 7-fold higher levels of copper compared to normal cells. The TPEN-copper complex undergoes redox cycling reactions. These results suggest that TPEN chelates accumulated copper in HCT116 cells making it available for redox cycling leading to cell toxicity and death. We further show that TPEN effectively inhibits colon cancer tumor growth in human colon cancer xenografts in mice. Therefore metal chelation provides a promising selective approach to target colon cancer.

## Methods

### Cell culture

Human colorectal cancer cells, SW480, HT-29 and LOVO were kindly provided by the American Type Culture Collection (ATCC). Cells were cultured in RPMI 1640 (Sigma-Aldrich, UK) with 20mM HEPES and L-Glutamine at 37°C in a humidified atmosphere of 5% CO2 and 95% air. Media was supplemented with 1% Penicillin-Streptomycin (100 U/ml) and 10% heat-inactivated FBS (Sigma-Aldrich, Germany). Unless otherwise mentioned, cells were seeded at 1.2 ×105 cells/ml and treated with TPEN (Sigma-Aldrich) at 50% confluence. TPEN was prepared in DMSO and the final DMSO concentration used on cells <0.3%.

### Cell viability assays & antibodies

Human HCT116 p53^+/+^ colon cancer cells were cultured as previously described [23]. Cell viability was measured using the MTT-based Cell Titer 96 non-radioactive cell proliferation kit (Promega Corp, Madison, Wisconsin, USA). Cell cycle analyses were performed on propidium iodide stained cells using flow cytometry (Becton Dickinson, Research Triangle, NC). The TUNEL assay used the *In Situ* Cell Death Detection Kit according to the manufacture instructions (Roche Diagnostics Corporation, Mannheim, Germany). For Annexin V staining cells were incubated in Annexin-V-Fluos labeling solution [20 μl Annexin reagent and 20 μl PI (50 μg/ml) in 1000 μl incubation buffer pH 7.4 (10 mM Hepes/NaOH, 140 mM NaCl, 5 mM CaCl_2_), then analyzed by flow cytometry. Caspase 3, 8 and 9 activities were assessed using Colorimetric Assay kits according to manufacturer insutructions (R & D Systems-BF4100). Primary antibody used for Western blots: XIAP #2042S; Caspase 3 #9665S; Caspase 9 #9502S; Bax #2772; PARP #9542S, from Cell Signaling. Cytochrome C sc-13560 from Santa-Cruz and GAPDH #5476 from Abnova.

### DCFH assay

Cells were treated with TPEN for 10, 20, 30 and 45 min. In experiments which involved addition of the antioxidant N-acetyl-L-cysteine (NAC), cells were treated with 5 mM NAC for 2 h before TPEN after which 10 μM of the CM-H2DCFDA dye was added for 20 min. Cells were washed, harvested by centrifugation and the pellet washed and re-suspended in 500 μl PBS followed by flow cytometery.

### Mitochondrial membrane potential

Cells were washed, pelleted and incubated in 500 μl of rhodamine buffer [5 μm rhodamine 123, 130 mM NaCl, 5 mM KCl, 1 mM Na_2_HPO_4_, 1 mM CaCl_2_, 1 mM CaCl_2_, 1 mM MgCl_2_, and 25 mM Hepes (pH7.4)] for 30 min at 37°C, then analyzed by flow cytometry.

### Atomic absorption

10^6^ HCT116 and NCM460 cells were collected in 8 ml HNO_3_ 65% + 2 ml H_2_O_2_ 30% and digested in a closed vessel microwave (Milestone ETHOS PLUS with HPR-1000/10S high pressure rotor). Cell lysates were measured for ion concentration against standard solutions prepared for Copper, Zinc and Iron in deionized water in an atomic absorption spectrophotometer (Furnace).

### UV/VIS and EPR spectroscopy

EPR spin-trapping experiments were carried out with a JEOL-RE1X spectrometer (Kyoto, Japan). Spectrometer settings were as follows: field center, 335.094 mT; microwave power, 10 mW; sweep time, 2.0 min; time constant, 0.3 s; and modulation width, 0.2 mT). UV/VIS spectra were recorded with Helios Alpha spectrophotometer (Thermo Fisher Scientific, Inc.; Pittsburgh, PA). All measurements were performed at room temperature.

### Mouse xenografts

NOD/SCID female mice, (6–8 weeks, ~20 g) (Charles River Laboratories, France) were divided into two groups of 8 mice each and maintained in Maxi-miser hepafiltered facility. 2-3×10^6^ HCT-116 tumor cells in 100 μl of 0.9% NaCl were inoculated subcutaneously in the flank. An average age (6–8 weeks) and body weight of mice (18-22 g) were used for the experiments. Prior to manipulations, mice were anaesthetized with isoflurane (Forane®, Abbott) by inhalation. On day 7 post inoculation mice received i.p. injections of either saline (control) or 20 mg/kg TPEN every other day for 28 days. Tumor measurements were performed 3 times per week using a sterile Vernier caliper. Tumor volume was calculated by the formula: Volume = π/6 (length × width × height). A 5-10% loss of body weight was detected in mice receiving TPEN treatment in comparison to the control but they otherwise looked healthy and tolerated the drug treatment well. The use of laboratory animals was in accordance with the guidelines of the Institutional Animal Care and Use Committee (IACUC). The IACUC committee at the American University of Beirut where the animal studies were conducted reviewed and approved the studies described.

### Immunohistochemical analysis of xenografts

Tissue sections (4 μm) were stained with and anti-Ki-67 antibody (Santa-Cruz, US), for 60 min followed by secondary and tertiary antibodies and incubated with the chromogen (Zymed, US) before counterstaining with Hematoxylin. To assess the extent of total cell death, tissue sections were stained by using the terminal deoxyribonucleotidyl transferase-mediated dUTP-nick-end labeling (TUNEL) assay, according to manufacturer’s instructions (in situ cell death detection kit, fluorescein; Roche) and counterstained with PI. Slides were analyzed under LSCM fluorescent confocal microscope (LSM 410, Zeiss, Germany).

### Statistical analyses

Data are expressed as the mean ± standard deviation, and statistical significance between different groups was determined using a two-tailed Student’s t-test. Statistical significance was defined as a *p < 0.05 and **p < 0.01.

## Results

### TPEN selectively kills HCT116 human colon cancer cells

**Figure 1:**
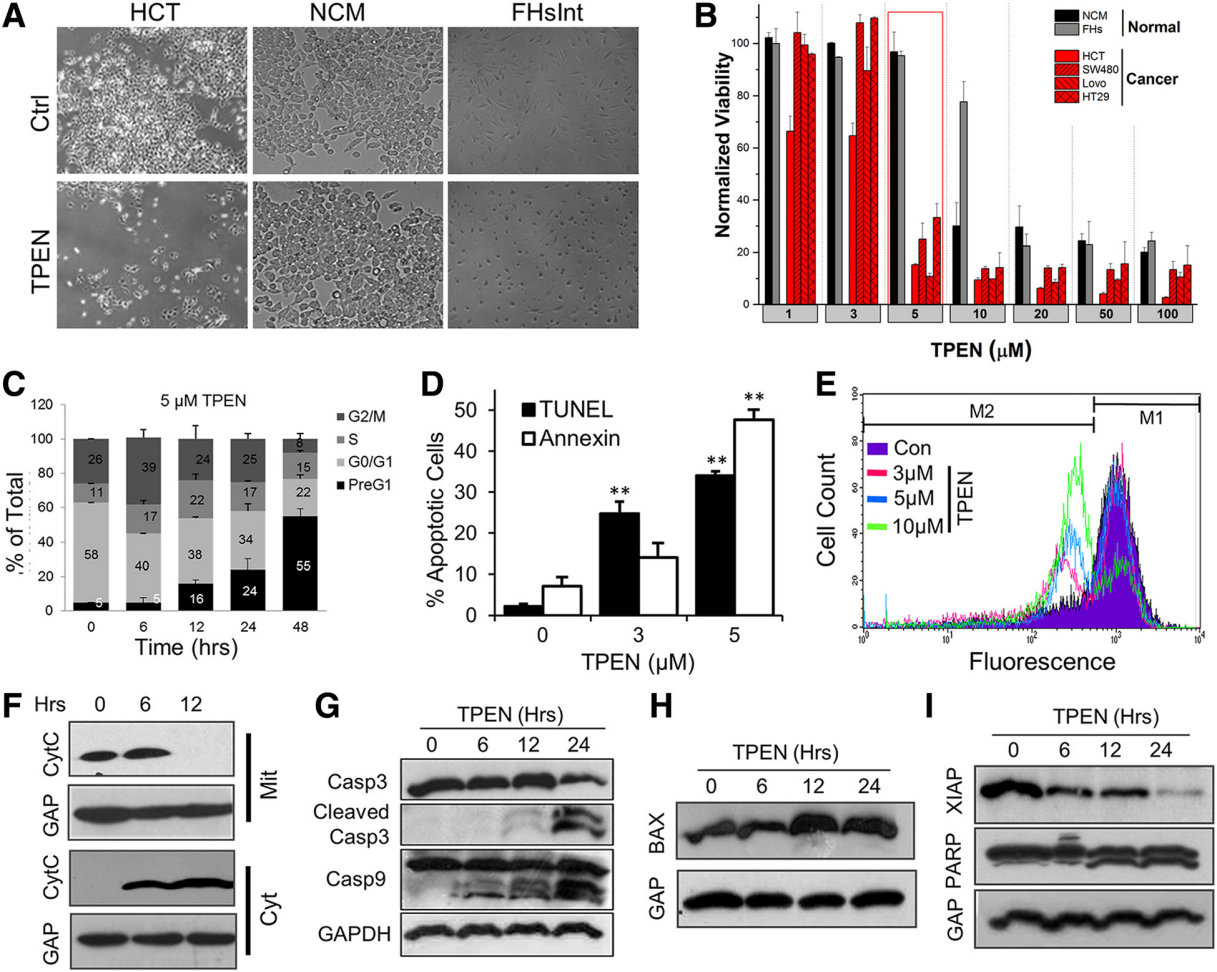
**TPEN selectively kills colon cancer cells. A)** Morphology of HCT116, NCM460, and FHS74Int cell lines with and without treatment with 5 μM TPEN for 24 h. **B)**Cell viability measured using the MTT assay as a function of TPEN concentrations in NCM 460, FHS74Int, HCT 116, SW480, LoVo and HT-29 cells (mean ± SD, n = 3). **C)** The mean percentage of cells in each cell cycle phase after DNA staining with PI and flow cyotmetry. **D)** TUNEL and Annexin assays in control cells and cells treated with TPEN 3 and 5 μM (mean ± SD, n = 3, ** p < 0.01, significant difference with respect to control). **E)** Rhodamine assay measuring disruption of mitochondrial membrane potential after TPEN treatment at 3, 5 and 10 μM (mean ± SD, n = 3). **F)** Time-dependent release of cytochrome c from mitochondria into the cytoplasm after TPEN treatment. **G**-**I)** TPEN alters the expression of several proteins involved in the apoptotic pathway in a time dependent manner. PARP, caspase-9 and caspase-3 were cleaved in response to TPEN. Bax levels were increased at 12 and 24 h of treatment.

Treatment of HCT116 colon cancer cells with 5 μM TPEN for 24 h results in a noticeable decrease in cell density (Figure 1A). In contrast, non-cancerous colon (NCM460) or intestinal (FHS74Int) cell lines (see methods for cell lines description) were resistant to TPEN at these concentrations (Figure 1A). A dose response of TPEN toxicity in the two non-cancerous (NCM460 and FHS74Int) and in four independent human colorectal cancer cell lines (HCT116, SW 480, HT-29, and LOVO), consistently revealed a significantly higher sensitivity of the cancerous cells as compared to their non-cancerous counterpart. The IC_50_ for the non-cancerous cells was 8.3 μM and 13μM for the NCM460 and FHS74Int respectively. In contrast the IC_50_ for the cancerous cells were 2.7; 4.0; 4.3 and 4.5 μM for the HCT116; SW480; HT-29 and LOVO cells respectively. Importantly, selective killing of cancerous cells (15.3 ± 0.4%; 25.07 + 6%; 10.82 + 1.18%; 33.35+ 5.22% survival for HCT, SW, LoVo, HT respectively) could be achieved at 5 μM TPEN for 24 h, concentrations at which 96.9 ± 7.52% and 95.3 ± 1.7% of the NCM460 and FHS74Int cell lines remained viable (Figure 1B). Flow cytometry analysis in HCT116 cells showed that TPEN toxicity is time-dependent as indicated by the gradual enrichment of the pre-G_1_ percentage in the cell population (Figure 1C). No obvious cell cycle arrest was associated with the TPEN treatment (Figure 1C), indicating that the primary effect of TPEN on HCT116 cells is to induce cell death.

### TPEN induces apoptosis in HCT116 cells

The observed increases in the PreG_1_ population are associated with higher levels of apoptosis, as indicated by TUNEL and Annexin V assays in TPEN treated cells in a dose-dependent fashion (Figure 1D). An early hallmark of most apoptotic cell death is increased mitochondrial membrane permeability. TPEN induced permeabilization of the mitochondrial membrane, as indicated by changes in mitochondrial membrane potential (ΔΨ_m_) (Figure 1E). Interestingly, this effect may be associated with TPEN’s ability to “extract” essential metals, particularly iron and copper from mitochondrial electron carriers, thus facilitating the loss of membrane potential and the deviation of electron flow to molecular oxygen O_2_ to generate superoxide and H_2_O_2_. Treatment with TPEN induced disruption of the mitochondrial membrane potential in a dose-dependent fashion, as detected by the reduced accumulation of the fluorescent dye rhodamine (Figure 1E). Consistently, disruption of the mitochondrial membrane is associated with the release of cytochrome c from the mitochondria and its accumulation in the cytosolic fraction (Figure 1F).

**Figure 2:**
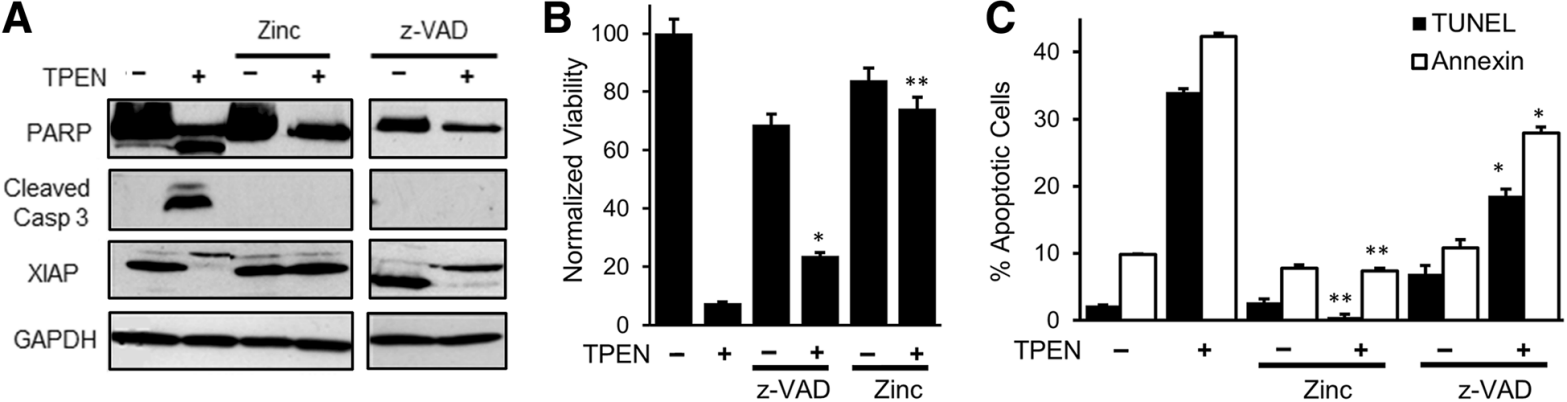
**TPEN**-**dependent cell death is partially due to apoptosis induction. A)** Cleavage of PARP, caspase-3 and degradation of XIAP in TPEN-treated cells was reversed by the addition of z-VAD-fmk or ZnSO_4_. **B)** MTT viability assay in control (-) and TPEN-treated (+) cell in the presence of either z-VAD-fmk to inhibit caspases or ZnSO_4_ (mean ± SD, n = 3). **C)** Annexin and TUNEL assays under similar condition as in panel **A)** (mean ± SD, n = 3). *p < 0.05 and ** p < 0.01, significant difference with respect to TPEN.

These results argue that TPEN induces apoptosis in HCT116 cells through the intrinsic pathway. In agreement with this conclusion, TPEN modulated key markers of apoptosis resulting in cleavage of caspases 3 and 9 into their active products (Figure 1G), cleavage of PARP, and accumulation of the pro-apoptotic BAX, which is typically observed upstream of mitochondrial dysfunction (Figure 1H). TPEN killing of cancer cells was reported to be associated with degradation of the X-linked inhibitor of apoptosis (XIAP) [15,24]. Consistently, treating HCT116 cells with TPEN results in XIAP degradation (Figure 2A, XIAP). XIAP degradation is blocked by incubations with zinc, showing that it is dependent on TPEN’s metal chelation properties (Figure 2A). However, inhibition of caspases with the pan-caspase inhibitor z-VAD was ineffective in blocking XIAP degradation (Figure 2C), arguing that TPEN-dependent XIAP degradation occurs upstream of caspase activation. These data show that TPEN induces apoptosis with all the associated molecular hallmarks.

The ability of TPEN to induce death of HCT116 colon cancer cells was dependent on its metal chelation properties, as it was reversed when TPEN was added in the presence of ZnSO_4_ (Figure 2B, Zinc). TPEN is a metal chelator with high affinity for zinc, copper and iron. Addition of exogenous Zn^2+^ is expected to associate with and saturate TPEN, preventing it from chelating other metals in the cell. ZnSO_4_ addition effectively abrogated the toxic effects of TPEN as indicated by the cell viability assay (Figure 2B, Zinc), TUNEL and annexin staining (Figure 2C, Zinc) and PARP, caspase 3 and XIAP cleavage (Figure 2A, Zinc). In contrast, inhibition of caspases using the pan-caspase inhibitor z-VAD-fmk resulted in significant, yet only partial reversal of TPEN toxicity (Figure 2B and 2C). z-VAD treatment effectively blocked caspase 3 and PARP cleavage (Figure 2A), this however was not sufficient to fully reverse TPEN-dependent cell killing (Figure 2B and 2C, z-VAD). These results suggest that the toxic effects of TPEN are mediated only partially through the induction of apoptosis, and potentially invoke other death pathways such as necrosis and autophagy.

### TPEN toxicity is associated with ROS generation

**Figure 3:**
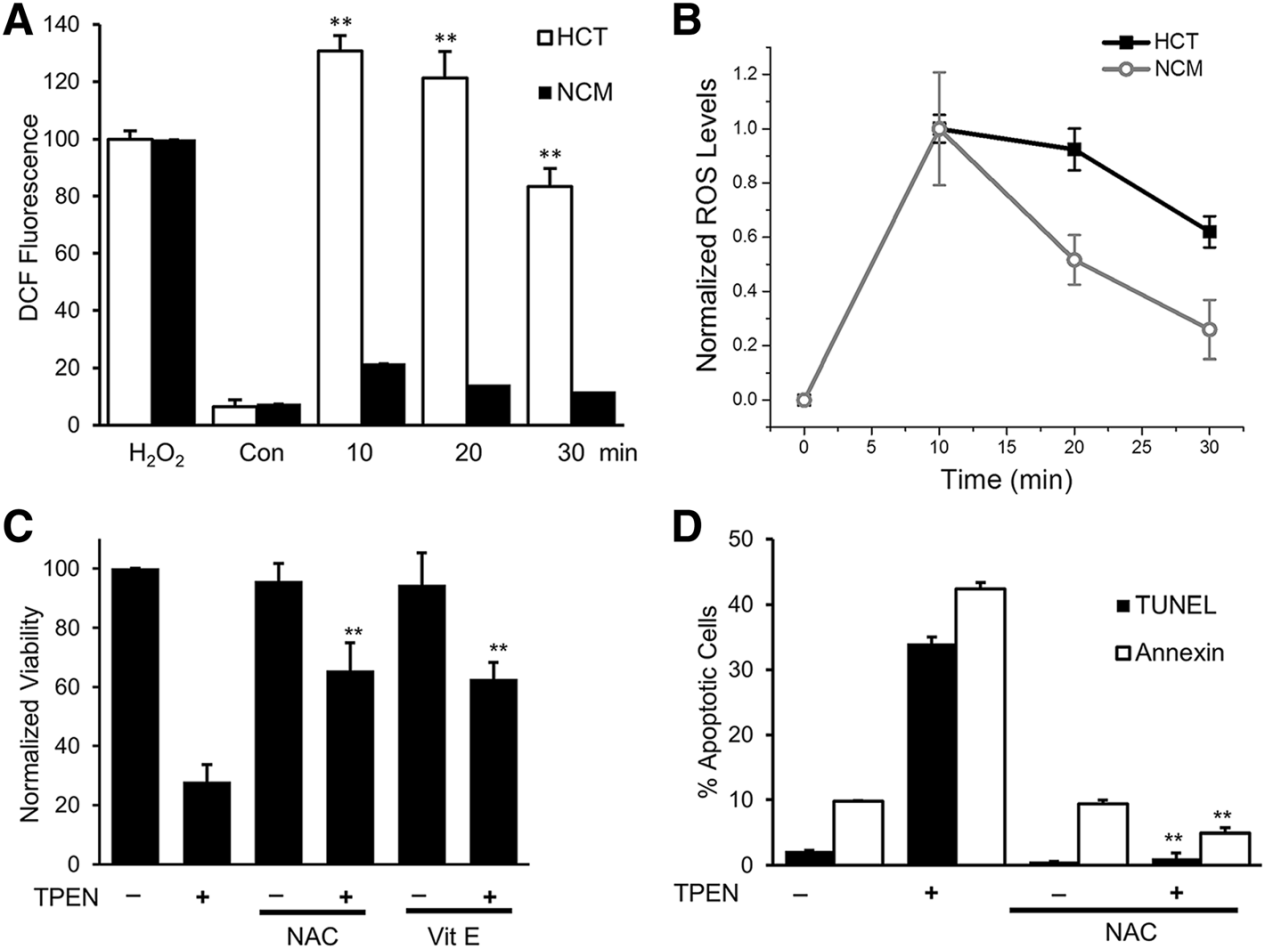
**TPEN induces ROS generation in HCT116 cancer cells. A)** Time course of TPEN-induced ROS generation in HCT116 and NCM460 cells. Control (Con) represents cells that have been treated with carrier DMSO. ROS generated in response to 250 μM H_2_O_2_ is also shown (mean ± SD, n = 3). **B)** Same data as in panel **A)** normalized to maximum levels of ROS induced in either the HCT116 or the NCM460 cells. Time zero represents carrier alone treatment and was set at zero. **C)** Cell viability in cells treated with TPEN in the presence or absence of the antioxidants NAC or vitamin E (mean ± SD, n = 3). **D)** Apoptosis measured using the TUNEL and Annexin assays in cells treated with TPEN in the presence and absence of NAC (mean ± SD, n = 3). ** p < 0.01, significant difference with respect to TPEN in **C**, and **D**, and with respect to control in **A**.

To investigate mechanisms involved in TPEN-dependent cell killing, we tested the involvement of reactive oxygen species (ROS) production using the DCFDA assay, since TPEN has been previously implicated in ROS production [25]. TPEN treatment caused a significant increase in intracellular ROS as early as 10 min after treatment, which gradually decreased over the next 30 min (Figure 3A). This ROS increase in HCT116 was significantly higher than that observed following the addition of 250 μM of H_2_O_2_ (Figure 3A). In contrast, TPEN-induced ROS production in the NCM460 normal colon cells was significantly smaller (Figure 3A), and decayed back to baseline with a faster time course than in HCT116 cells (Figure 3B). Similar to the data obtained in HCT116 cells, TPEN treatment of two other colon cancer cell lines, SW480 and LOVO, also resulted in the production of significantly higher levels of ROS as compared to the normal NCM colon cells (Additional file 1: Figure S1). These data show that TPEN induces a 6-7fold higher ROS in colon cancer cells as compared to the normal NCM460 cells, and that this increased ROS lasts for tens of minutes longer in HCT116 cells (Figure 3A, 3B and Additional file 1: Figure S1). Hence there is good correlation between ROS induction and TPEN toxicity in these two cell lines.

To assess whether ROS generation is involved in mediating TPEN-induced cell death, we pretreated HCT116 cells with antioxidants, including NAC and vitamin E, after which cell viability and apoptosis were assessed. As expected, the general ROS scavenger NAC and vitamin E significantly decreased H_2_O_2_- and TPEN-induced ROS generation (Additional file 2: Figure S2). Antioxidants effectively prevented TPEN toxicity as measured by cell viability (Figure 3C), TUNEL and annexin assays (Figure 3D), and reversed caspase 3 and 9 activation (Additional file 3: Figure S3). This argues that TPEN-mediated cell killing requires ROS production. Furthermore, the protective effects of antioxidants are also compatible with the idea of transition metal catalyzed oxidative stress. However, the observed incomplete protection by two different antioxidants – NAC and Vit E – may indicate contribution of non-redox driven pathways, such as Zn-dependent effects, given previous reports that argued for a role of Zn-homeostasis in TPEN toxicity. Nonetheless, the significant reversal of the TPEN toxicity using two different anti-oxidants strongly argues for an essential role for ROS production in TPEN toxicity.

### TPEN-metal complex are redox active

**Figure 4:**
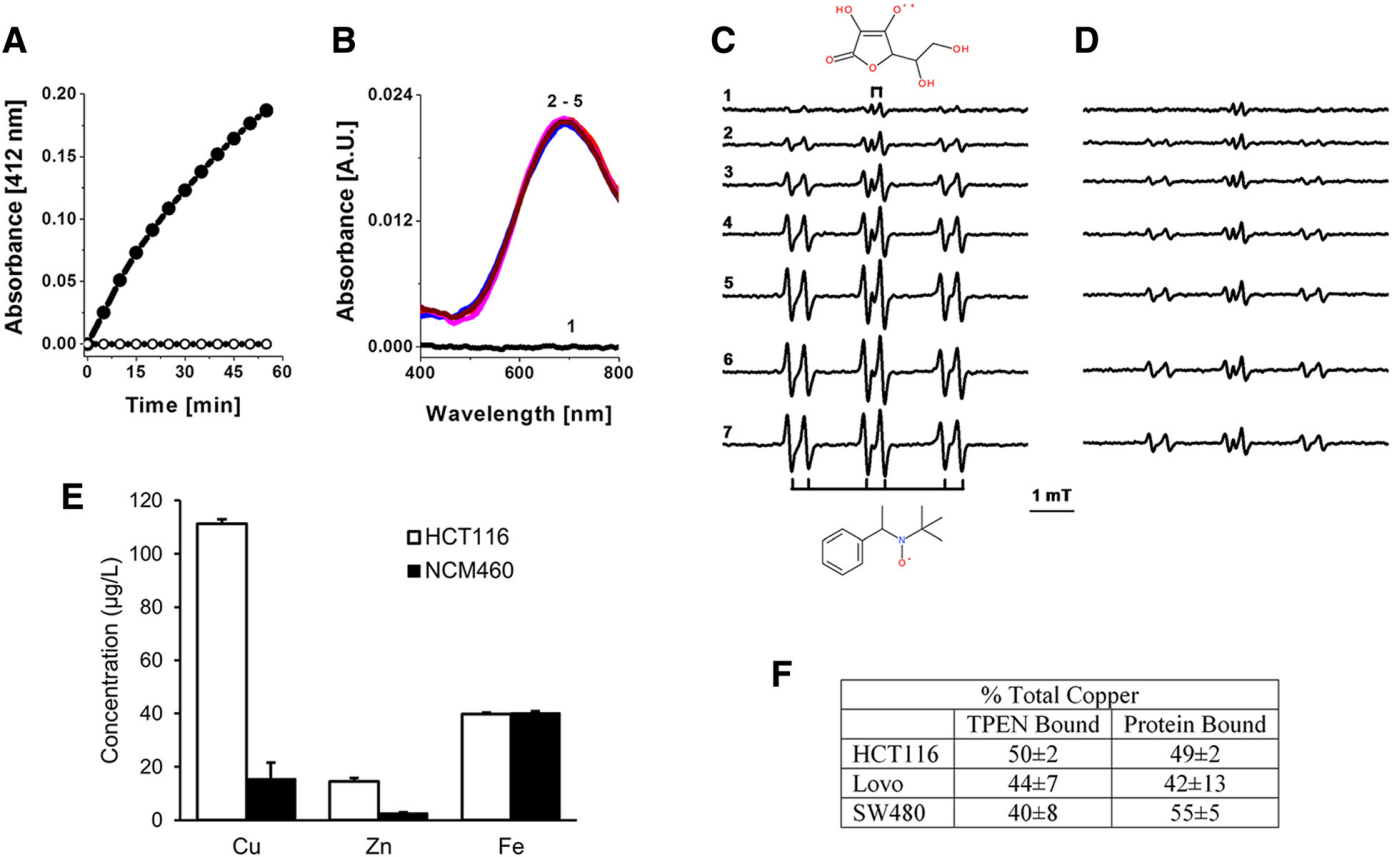
**TPEN**-**Cu complex is redox active. A**) Reduction of [TPEN-Fe ^(III)^] and [TPEN-Cu ^(II)^] by ascorbic acid. Reactions were carried out at 25°C in 0.1 M phosphate buffer (pH 7.4). TPEN, ferric nitrate, copper sulfate and ascorbic acid were used at concentrations of 0.2, 0.1, 0.1 and 5 mM, respectively. Complexes of TPEN with Cu ^(II)^ and Fe ^(III)^ were prepared via incubation of the ligand (0.3 mM) with the corresponding metal ion (0.03 mM) for 10 min in the reaction buffer. **A)** Changes in the absorbance of solutions of [TPEN-Fe^(III)^] in the absence (open circles) and the presence (closed circles) of ascorbic acid. **B)** Electronic spectrum of TPEN (1) and [TPEN-Cu^(II)^] in the absence (2) and the presence (3 – 5) of ascorbic acid. Consecutive spectra were recorded with time interval of 5 min. **C**-**D)** Fenton-like reactions of [TPEN-Cu^(II)^] and [TPEN-Fe^(III)^]. **C)** [TPEN-Cu^(II)^], ascorbic acid, DMSO, H_2_O_2_ and PBN. **D**) [TPEN-Fe^(III)^], ascorbic acid, DMSO, H_2_O_2_ and PBN. Reactions were carried out 25°C in 0.1 M phosphate buffer (pH 7.4). Ascorbic acid, DMSO, H_2_O_2_ and PBN were used at concentrations of 1 mM, 300 mM and 2 mM, respectively. Consecutive EPR spectra were recorded with time interval of 5 min. **E)** Concentrations of copper, zinc and iron in HCT116 and NCM460 (mean ± SD, n = 3). **F)** Percent copper chelated by TPEN in HCT116, Lovo and SW480 cells. Cells were treated with 5 μM TPEN for 24 h, and prepared cell lysates were filtered through 10 Kd cut off filtered to remove TPEN and TPEN-copper complexes. Copper was measured by atomic absorption in both the filtrate (TPEN Bound) and the lysates (Protein Bound) and percentages calculated accordingly (mean ± SD, n = 3).

We were then interested in defining the mechanism by which TPEN induces ROS generation and cell killing. In the presence of ascorbic acid, TPEN has been shown to mobilize iron from ferritin, presumably via formation of a [TPEN-Fe^(II)^] complex. This reaction may have toxicological consequences, as [TPEN-Fe^(II)^] can participate in Fenton-like reactions with concomitant generation of hydroxyl radical and/or high-valent iron ^(IV)^-oxo species. In addition, TPEN was suggested to deplete intracellular copper in several cell types [11,26,27]. Hence, we carried out experiments to verify whether ascorbic acid can reduce [TPEN-Fe^(III)^] to [TPEN-Fe^(II)^], and [TPEN-Cu^(II)^] to [TPEN-Cu^(I)^]. This is important in the context of the ROS observed in response to TPEN treatment because the reduced forms of these complexes may have the potential to generate hydroxyl radical in the presence of H_2_O_2_.Ascorbic acid reduces [TPEN-Fe(III)] at a relatively low rate (Figure 4A, closed circles), thus setting the stage for induction of a futile redox cycle whereby [TPEN-Fe(II)] autoxidizes to [TPEN-Fe(III)] with concomitant generation of superoxide anion radical. Incubation of ascorbic acid with [TPEN-Cu(II)], however, did not result in changes in the electronic spectrum of the complex (Figure 4B). This suggests that either ascorbate does not possess the required potential to reduce [TPEN-Cu(II)] to [TPEN-Cu(I)], or the latter rapidly autoxidized with generation of superoxide anion radical. To verify the latter hypothesis, we assessed the ability of [TPEN-Cu(II)] to generate hydroxyl radical in a reaction system consisting of H2O2, ascorbic acid, dimethylsulfoxide (CH3SOCH3; DMSO) and N-tert-butyl-alha-phenylnitrone (PBN) as a spin trapping agent. In the complete reaction system, the initial Electron Paramagnetic Resonance.

(EPR) doublet of the semidehydroascorbyl radical (ascorbate + radicals → semidehydroascorbyl radical) was gradually substituted by the characteristic 6-line EPR spectrum of PBN/^.^CH_3_ (Figure 4C; CH_3_SOCH_3_ + HO^.^ → ^.^CH_3_ + CH_3_S (O) OH; ^.^CH_3_ + PBN → PBN/^.^CH_3_^.^). The formation of PBN/^.^CH_3_ was inhibited by depletion of ascorbic acid, as assessed by the disappearance of the semidehydroascorbyl radical. No PBN/^.^CH_3_ formation was observed if either H_2_O_2_ or ascorbic acid was omitted from the reaction system. Under these experimental conditions, the rate of PBN/^.^CH_3_ formation by [TPEN-Fe^(III)^] was 4 times slower than that observed with [TPEN-Cu ^(II)^] (Figure 4D). The results obtained indicate that both [TPEN-Fe^(II)^] and [TPEN-Cu ^(I)^] have the potential to generate HO^.^ in biological systems ([TPEN-Me^(n+)^] + H_2_O_2_ → [TPEN-Me^(n+1)^] + HO^.^ + HO^-^). These observations extend the reaction mechanism of the breakdown of H_2_O_2_ by ([TPEN-Fe^(n+)^], which includes the formation of high-valent oxo-species that can activate C-H bonds and epoxidate > C = C < bonds [28-30]. Hence the TPEN-Fe and TPEN-Cu complex engage in redox cycling resulting in the generation of ROS. As such chelation of cellular copper and/or iron by TPEN has the potential to produce ROS leading to cellular toxicity.

### Colon cancer cells accumulate higher levels of copper and zinc

Given the potential of TPEN-Cu ^(II)^ and TPEN-Fe^(II)^ to redox-cycle in the presence of H_2_O_2_ and a reductant (eg, ascorbate) to yield hydroxyl radicals, the ability of these TPEN-metal complexes to produce cellular damage is likely to be proportional to the amount of chelatable metals in cells. Tumor tissues from multiple cancers have been reported to have elevated copper content [22]. This is also the case for HCT116 cells as shown by atomic absorption spectroscopy studies, where both copper (enriched ~7-folds) and zinc (enriched ~5.5-folds) accumulate to significantly higher levels as compared to NCM460 (Figure 4E). In contrast, iron content was similar between the two cell types (Figure 4E). Similar accumulation of copper and zinc but not iron were observed in two additional colon cancer cell lines, LoVo and SW480, where the measured levels for Cu were 120.25 and 99.56 μg/L; Zn 20.34 and 15.26 μg/L; Fe 44.24 and 46.25 μg/L in LoVo and SW480 cells respectively. Given that zinc is not redox active and that the levels of iron are similar in both cell types, the mechanisms of action of TPEN is likely to involve formation of a TPEN-Cu complex that engages in redox cycling to generate ROS and cause cellular damage. Interestingly the fold enrichment of copper in HCT116 cells (Figure 4E) mirrors the fold increase in redox-cycling activity revealed by DCFH oxidation measured in these cells as compared to NCM460 following TPEN treatment (Figure 3A).

### Cellular copper is required for TPEN toxicity

The above data support a model where TPEN induces selective death of cancer cells through its ability to chelate copper away from cellular proteins, resulting in the formation of a TPEN-copper complex able to engage in redox cycling thus generating ROS and inducing cell death. Interestingly, the TPEN-Cu complex is predicted to be impermeant to the cell membrane, given the positive charges contributed by the copper ion, thus trapping the complex intracellularly and increasing the levels of ROS produced. This model makes two testable predictions. First, it predicts that TPEN is capable of stripping a significant proportion of copper away from cellular proteins. To determine whether this is the case, we measured the fraction of total cellular copper that is chelatable by TPEN under our experimental conditions. As shown in Figure 4F, ~50% of the total cellular copper can be chelated by TPEN following incubation with 5μM TPEN for 24 hrs.

**Figure 5:**
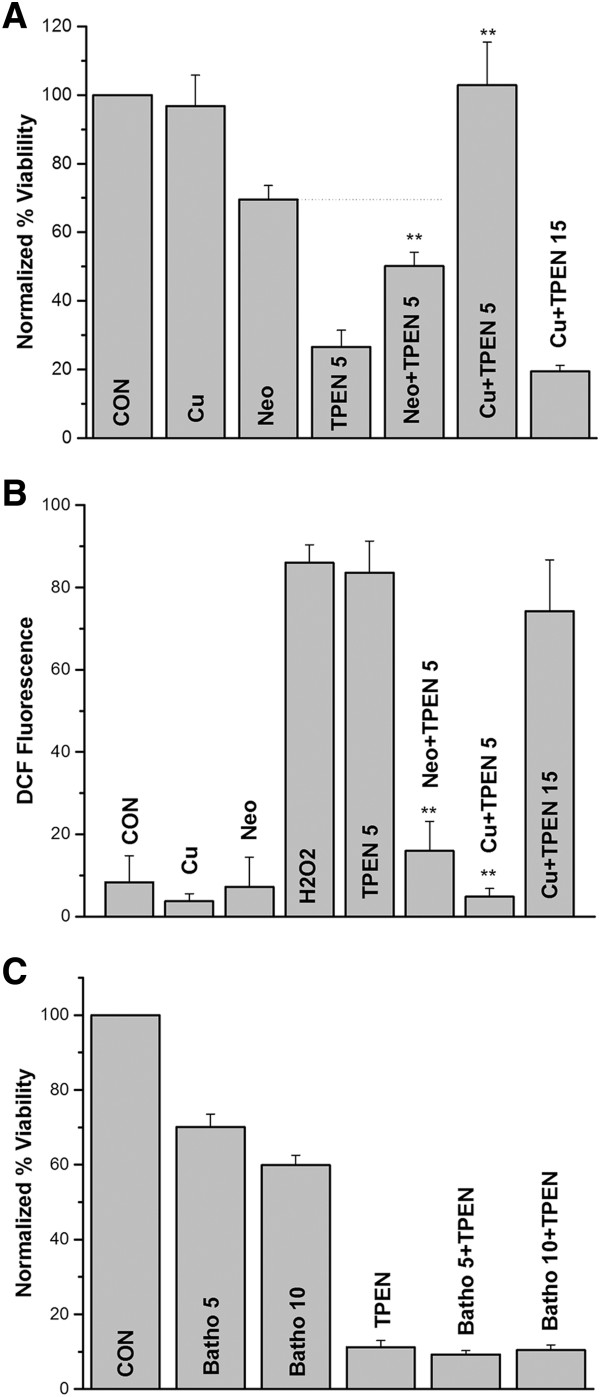
**Cellular Cu is required for TPEN toxicity. A)** Cellular viability using the MTT assay in cells that were either untreated (CON), incubated with CuSO_4_ (5 μM, Cu); neocuproine (25 μM, Neo); TPEN (5 μM); pre-incubated with neocuproine (25 μM) for 2 h before TPEN (5 μM) addition (Neo + TPEN); pre-incubated with CuSO_4_ (5 μM) before TPEN (5 μM, Cu + TPEN5) or TPEN (15 μM, Cu + TPEN15) addition (mean ± SD, n = 3). **B)** ROS induction using the DCF assay in untreated cells (CON), cells incubated with CuSO_4_ (5 μM, Cu); neocuproine (25 μM, Neo); H_2_O_2_ (250 μM); TPEN (5 μM); pre-incubated with neocuproine (25 μM) for 2 h before TPEN (5 μM) addition (Neo + TPEN); pre-incubated with CuSO_4_ (5 μM) before TPEN (5 μM, Cu + TPEN5) or TPEN (15 μM, Cu + TPEN15) addition (mean ± SD, n = 3). **C)** MTT viability assay in untreated cells (CON); cells incubated with bathocuproine ( 5 and 10 μM) or TPEN (5 μM), and in cells pre-incubated with bathocuproine (5 or 10 μM) for 2 hours before the addition of TPEN (5μM) (mean ± SD, n = 3, ** p < 0.01, significant difference with respect to TPEN in **A** and **B)**.

The second prediction from the proposed model is that cellular copper is essential for TPEN toxicity. Indeed if TPEN induces cell death through redox cycling after the formation of a TPEN-Cu complex, then reducing the enriched total cellular copper in cancerous HCT116 cells would be expected to reduce TPEN toxicity. To test whether this is the case we incubated HCT116 with the membrane permeant copper specific chelator neocuproine [31]. We chose neocuproine because of its specificity for copper and because the neocuproine-Cu complex in unlikely to be redox active [32]. Therefore, preincubation of HCT116 cells with neocuproine is expected to chelate cellular copper thus preventing TPEN from binding it. In the absence of the formation of TPEN-Cu complexes TPEN treatment should not produce ROS. Neocuproine by itself mildly reduces cellular viability (Figure 5A, Neo), probably due to the requirement for copper for normal cellular homeostasis. Importantly, incubating cells with neocuproine does not generate ROS (Figure 5B, Neo), showing that indeed the Neo-Cu complex is not redox active. However, pre-incubating HCT116 cells with neocuproine significantly reverses the toxic effect of TPEN (Figure 5A, Neo + TPEN), and this reduction in toxicity is coupled to a significant reduction in the levels of ROS generated (Figure 5B, Neo + TPEN5).

To test whether neocuproine reverses TPEN toxicity by chelating intracellular copper, we incubated cells with a membrane impermeant copper specific chelator, bathocuproine [33]. In contrast to neocuproine, bathocuproine did not reverse the cellular toxicity due to TPEN treatment (Figure 5C). Collectively these data show that TPEN toxicity requires cellular copper. When intracellular copper is chelated with neocuproine, a copper chelator that is effective at chelating copper but does not allow the TPEN-Cu complex to engage in redox reactions, TPEN becomes ineffective at producing ROS and killing colon cancer cells. Furthermore, as shown in Figure 2A with zinc, copper was also effective at reversing the toxic effects of TPEN and its ability to generate ROS in a dose dependent fashion (Figure 5A and B), showing that TPEN toxicity is dependent on its metal chelation properties.

### Tumoristatic activity of TPEN in vivo

**Figure 6:**
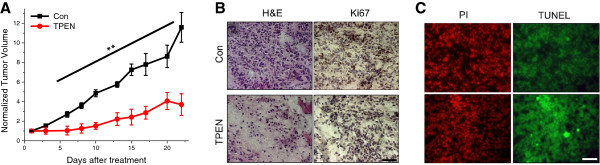
**TPEN decreases the volume of colon cancer xenografts in mice. A)** Graph showing a significant decrease in the volume of HCT116 colon cancer xenografts in mice injected with 20 mg/kg TPEN i.p., three times per week for 22 days. Tumor volumes were significantly smaller in TPEN-treated mice as compared to saline injected (Con) starting at day 6 after treatment (mean ± SEM, n = 7-8, ** p < 0.01, significant difference with respect to Con in **A)**. **B)** Histological (H & E) and immunohistochemical (Ki-67 antibody) staining in tumor xenografts. **C)** Apoptosis staining is revealed by using the TUNEL assay. Tissue was processed as described in “Methods.” Identical fields are shown for propidium iodide (PI) stain of all nuclei (red fluorescence), and for fluorescein-labeled detection of TUNEL-positive nuclei (green fluorescence). Representative images were taken at 400× magnification. Scale Bar, 50 μm.

The ability of TPEN to selectively kill cancer cells raises the attractive possibility that it could have anti-tumor potential *in vivo*. To test whether this is the case we used a mouse xenograft colon cancer model. HCT116 colon cancer cells (2-3×10^6^), were subcutaneously inoculated into the flank of NOD/SCID mice, and showed efficient engraftment as reflected by the development of malignant palpable tumor mass within 7–9 days post-inoculation. To investigate the therapeutic efficacy of TPEN, xenografted mice were injected intraperitoneally (i.p.) at 7 days post-inoculation with TPEN (20 mg/kg) every other day for a 22 days therapy span. Tumor size was monitored in mice at different time points starting at day 7 post inoculation for both groups. In the TPEN treated group tumor volume was significantly reduced throughout the time course of tumor development with a volume of 11.6 ± 0.37 cm^3^ in the control group versus 3.75 ± 0.142 cm^3^ in the TPEN-treated group at day 22 (Figure 6A). In our hands TPEN was well tolerated at 15–20 mg/kg, but not at 30 mg/kg, consistent with previous reports [34].

Histological H & E staining of tumor sections from untreated mice showed malignant cell infiltration and retention in the tissue (Figure 6B). However, TPEN-treated group showed a significant decrease of malignant cell nuclei staining relative to the control mice, consistent with the tumor growth profile (Figure 6A and B). In addition, the expression of Ki-67, a nuclear protein proliferative marker, was reduced in TPEN-tissue section as depicted by immunostaining (Figure 6B). Furthermore, in vivo TPEN treatment induced apoptosis in tumor tissue as reported by TUNEL staining (Figure 6C). Collectively these data show that TPEN is effective at inhibiting the growth of colon cancer cells *in vivo*, arguing that metal chelation is a promising intervention for colon cancer treatment.

## Discussion

Metals such as iron, copper and zinc are essential elements in mammalian cells and are used as cofactors or as structural components of many enzymes. However, an excess of these metals causes toxicity. Therefore a balance between metal accumulation, their sequestration within cellular compartments, and their association with cellular proteins is essential for the maintenance of cell viability [35,36]. Transient intracellular elevations of free iron or copper is toxic because of their redox reactivity and participation in ROS metabolism. In particular, low molecular mass complexes of Fe ^(II)^ and Cu ^(II)^ can react with hydrogen peroxide to generate hydroxyl radical, which is believed to be the most toxic form of ROS encountered in cells.

Here we show that this detrimental effect of transition metals can be used effectively to selectively eliminate cancer cells. TPEN is efficient at selectively eliminating colon cancer cells both in the dish and *in vivo* in mice. The mechanism of action of TPEN depends first on its high affinity for metals and as such its ability to strip intracellular metals from cellular proteins. Second colon cancer cells accumulate copper to significantly higher levels than normal colon cells. Finally, the TPEN-copper complex engages in redox cycling and the generation of ROS (hydroxyl radicals). Hence, TPEN selectively eliminates colon cancer cells using an elegant mechanism that relies on the biology of the cancer through its tendency to accumulate copper, a feature that is common to many other cancers including breast, ovarian, stomach, colorectal and leukemia [22]. As such the TPEN-dependent selective killing observed in this study is likely to be applicable to other cancers that accumulate redox active metals.

However, TPEN anti-tumor activity is likely to involve additional mechanisms since antioxidants treatment was not fully effective at reversing TPEN toxicity (Figure 3). TPEN toxicity is likely to be partly due to the removal of zinc from essential Zn-dependent proteins as previously reported by others [7-15].

Another metal chelator that shows selective antitumor activity is di-2-pyridylk-etone-4,4,-dimethyl-3-thiosemicarbazone (Dp44mT). Dp44mT is thought to act in a similar fashion to TPEN through iron chelation and redox cycling to generate ROS [37,38]. Dp44mT toxicity is also believed to involve activation of the lysosomal apoptotic pathway through its copper binding capacity [20].

Interestingly, cancer cells are known to have an altered redox status with an up-regulation of oxidative stress and an augmentation of antioxidant capacity (reviewed in [39]). It is also thought that a moderate increase in ROS enhances cell survival and proliferation [40]. Therefore the observed increase in ROS in cancer cells may promote tumorigenesis [39]. However, the increased basal ROS in cancer cells brings them closer to the toxicity threshold where the intrinsic antioxidant capacity, although enhanced, is not sufficient to contain toxic ROS levels [39]. Therefore increased oxidative stress in cancer cells represents an effective mechanism to eliminate cancer cells [39].

In summary, selective killing of colon cancer cells can be achieved using TPEN, where the transition metal-chelator complex engages in redox cycling and the generation of hydroxyl radicals. This is an attractive potential anti-tumor therapeutic approach because its mechanism of action depends on the biology of cancer cells, including the significant accumulation of copper and their endogenous enhanced oxidative stress. Therefore, selectivity toward elimination of cancer cells without affecting normal cells is inherent in this approach because of different homeostatic mechanisms in cancer versus normal cells. In that context it would be attractive to systematically explore the redox cycling potential of metal chelators already in the clinic for the treatment of other diseases.

## Conclusions

TPEN, a membrane permeant metal chelator, selectively kills colon cancer cells without affecting the viability of normal cells. TPEN is effective at preventing tumor growth in a xenograft model. The mechanism of action of TPEN involves chelation of intracellular copper, making it available for redox cycling thus leading to the generation of ROS and cell death. Because colon cancer cells accumulate copper to 7 folds higher levels than control cells, this endows TPEN with its selective killing ability. Therefore, the mechanism of action of TPEN offers an attractive anti-tumor therapeutic approach potentially for a multitude of cancers that accumulate copper.

## Abbreviations

DCFDA: 2 ′,7′-Dichlorofluorescin diacetate; DMSO: Dimethyl sulfoxide; Dp44mt: Di-2-pyridylketone-4,4,-dimethyl-3-thiosemicarbazone; NAC: N-Acetylcysteine; Neo: Neocuproine; PARP: Poly aDP ribose polymerase; PBN: N-tert-butyl-alha-phenylnitrone; ROS: Reactive oxygen species; TPEN: *N*, *N*, *N*’, *N*’-tetrakis-[2-pyridylmethyl]-ethylenediamine; TUNEL: Terminal deoxynucleotidyl transferase dUTP nick end labeling; XIAP: X-Linked inhibitor of apoptosis.

## Competing interests

The authors declare that they have no competing interests.

## Authors’ contributions

MF, RAM, and DAS designed and performed experiments and analyzed data; OR, AZ, and HH performed experiments; VEK, HGM and KM designed experiments and analyzed data; MF, HGM and KM wrote the paper. All authors read and approved the final manuscript.

## Pre-publication history

The pre-publication history for this paper can be accessed here:

http://www.biomedcentral.com/1471-2407/14/527/prepub

## Supplementary Material

Additional file 1: Figure S1 TPEN induces high ROS levels in cancerous but not normal colon cells. Time course of TPEN-induced ROS generation in LoVo, SW480 and NCM460 cells. Control (Ctrl) represents cells that have been treated with DMSO carrier alone. ROS generated in response to 250 mM H2O2 is also shown as a control (mean ± SD, n = 3). ** p < 0.01, significant difference with respect to Ctrl. Click here for file

Additional file 2: Figure S2 Antioxidants prevent TPEN-induced ROS generation in HCT116 cancer cells. Pretreatment with the antioxidants NAC or vitamin E decreased ROS production in cells treated with TPEN. ROS generated in response to 250mM H2O2 is also shown as a control (mean ± SD, n = 3). **p < 0.01, significant difference with respect to TPEN. Click here for file

Additional file 3: Figure S3 Effect of TPEN on caspase activity. TPEN treatment (TPEN) significantly increased caspase-3 **(A)** and caspase-9 **(B)** above baseline (Ctrl). Pre-treatment with the antioxidant NAC decreases caspase-3 **(A)** and caspase-9 **(B)** activity below baseline levels at 12 h. Click here for file
