# Supplementary figures and images for: Copper chelation selectively kills colon cancer cells through redox cycling and generation of reactive oxygen species
Source: BMC Cancer. 2014 Jul 21;14:527. doi: 10.1186/1471-2407-14-527 (PMC4223620; doi:10.1186/1471-2407-14-527)

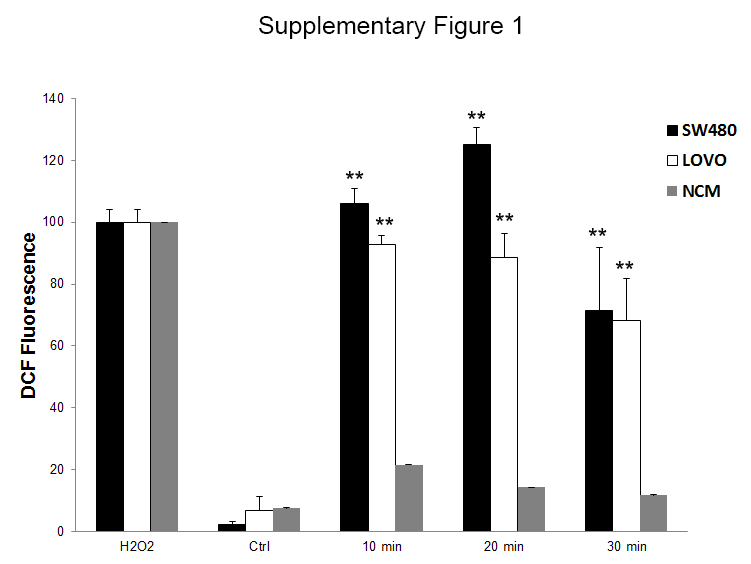

Supplement: Additional file 1: Figure S1 — TPEN induces high ROS levels in cancerous but not normal colon cells. Time course of TPEN-induced ROS generation in LoVo, SW480 and NCM460 cells. Control (Ctrl) represents cells that have been treated with DMSO carrier alone. ROS generated in response to 250 mM H2O2 is also shown as a control (mean ± SD, n = 3). ** p < 0.01, significant difference with respect to Ctrl. [file 1471-2407-14-527-S1.tiff]

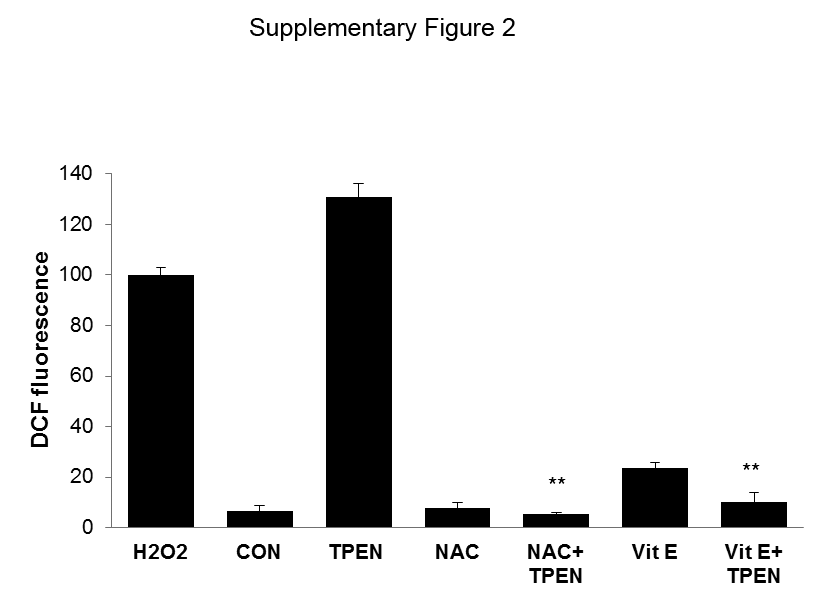

Supplement: Additional file 2: Figure S2 — Antioxidants prevent TPEN-induced ROS generation in HCT116 cancer cells. Pretreatment with the antioxidants NAC or vitamin E decreased ROS production in cells treated with TPEN. ROS generated in response to 250mM H2O2 is also shown as a control (mean ± SD, n = 3). **p < 0.01, significant difference with respect to TPEN. [file 1471-2407-14-527-S2.tiff]

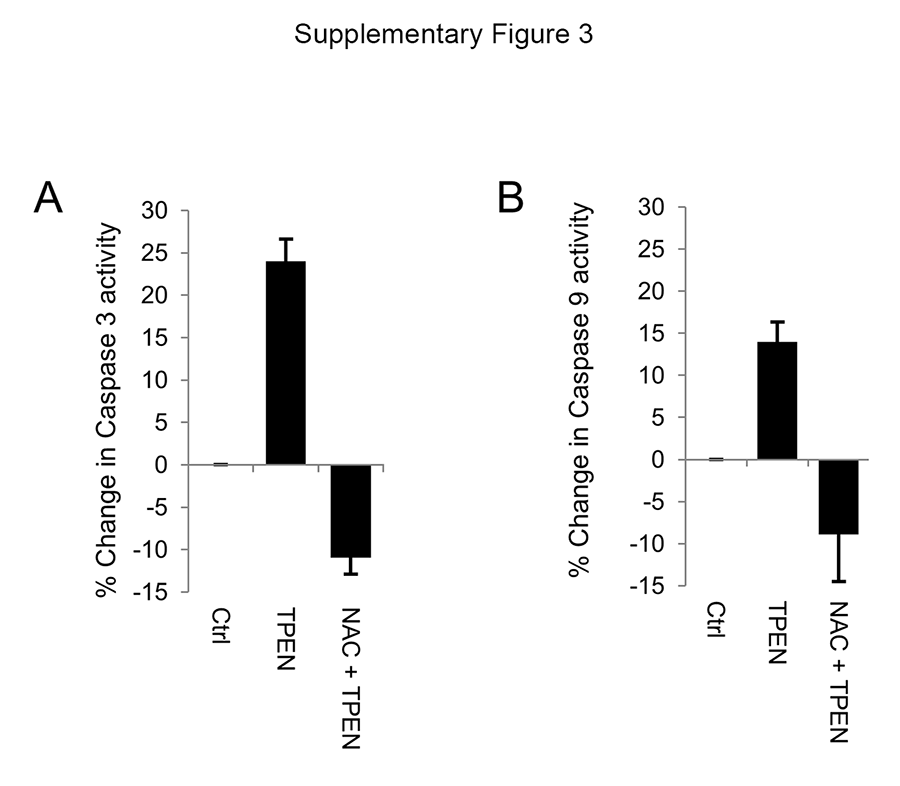

Supplement: Additional file 3: Figure S3 — Effect of TPEN on caspase activity. TPEN treatment (TPEN) significantly increased caspase-3 (A) and caspase-9 (B) above baseline (Ctrl). Pre-treatment with the antioxidant NAC decreases caspase-3 (A) and caspase-9 (B) activity below baseline levels at 12 h. [file 1471-2407-14-527-S3.tiff]
